# Comparative evaluation of sealing ability, penetration and adaptation of a self etching pit and fissure sealant- stereomicroscopic and scanning electron microscopic analyses

**DOI:** 10.4317/jced.55624

**Published:** 2019-06-01

**Authors:** Dhruv Garg, Karuna Mahabala, Amita Lewis, Srikant Natarajan, Anupama Nayak, Ashwin Rao

**Affiliations:** 1 Former student. Manipal College of Dental Sciences, Mangalore, Manipal Academy of Higher Education, Manipal; 2 Assistant Professor. Department of Pedodontics and Preventive Dentistry, Mangalore, Manipal Academy of Higher Education, Manipal; 3 Reader. Department of Oral Pathology and Microbiology, Mangalore, Manipal Academy of Higher Education, Manipal; 4 Professor and Head. Department of Oral Pathology and Microbiology, Mangalore, Manipal Academy of Higher Education, Manipal; 5 Associate Professor. Department of Pedodontics and Preventive Dentistry, Mangalore, Manipal Academy of Higher Education, Manipal

## Abstract

**Background:**

The efficacy of pit and fissure sealants in preventing occlusal caries is a well-established fact. Considering the difficulty in achieving strict isolation for a longer duration while treating the pediatric patients, a simplified procedure of sealant application is desirable. While, a self-etching sealant, Prevent Seal offers a quick procedure, the physical properties of this material haven’t been studied yet. Thus, this study was aimed to comparatively evaluate sealing ability, penetration and adaptation of a self-etching pit and fissure sealant and a conventional resin sealant.

**Material and Methods:**

This was an *in vitro*intergroup comparative study, which consisted of 2 groups- Group I (Conventional acid etch sealant, Clinpro) and Group II (Self etching sealant, Prevent Seal). Out of 32 selected teeth 16 were used to study microleakage, with the help of dye penetration test using Övrebö and Raadal criteria. Remaining 16 were used to evaluate sealant penetration and adaptation viz bubbles in the bottom of fissure, debris in the fissure, tags in the bottom of the fissure and tags at cuspal slopes and fissure entrance was done using stereomicroscope. Post stereomicroscopic evaluation 4 samples each were randomly chosen from both the groups and checked for etching pattern using Scanning electronic microscope.

**Results:**

The comparison of tested properties between the groups was done using Chi square test. There was no statistically significant difference observed when microleakage and sealant penetration / adaptation properties were compared between two groups (*p*=0.63 and *p*= 0.131, 0.131, 0.302, 0.106 respectively). No conclusive results could be withdrawn while etching patterns were compared between the groups (*p*=0.717).

**Conclusions:**

The self-etching sealant Prevent seal was found to have similar microleakage, sealant penetration and adaptation properties as conventional acid etch sealant.

** Key words:**Pit and fissure sealant, self-etching, scanning electron microscope, sealing ability.

## Introduction

The efficacy of pit and fissure sealants in preventing occlusal caries, especially in individuals with high caries risk is time tested and strongly literature supported (1-4). However, the success rate of sealants is mainly dependent on their ability to prevent the accumulation of biofilm and their acidic byproducts (5). Thus, adhesion of the sealant on to the tooth surface is very critical for the procedure to be a successful one. Etching the tooth enamel with phosphoric acid is the conventional and standard technique practiced over a period of time to help resin sealants to adhere firmly on to the intended pits and fissures (6).

Etching is a very critical step during any adhesive procedure to achieve a good bond (1). The bond strength between enamel and resin depends on obtaining an etching pattern, that facilitates the formation of resin tags (7). Salivary contamination post etching the enamel, decreases the adhesion of the sealant due to the formation of a surface coating, thus requiring the etching procedure to be repeated all over again (6,8). Achieving strict isolation for a longer duration is a difficult task while treating the pediatric patients. Thus, the procedure of application of sealant that is quick and simple is need of the hour (6).

Introduction of “all-in-one” system has made adhesive dentistry simple and promising by offering reliable bonding to both enamel and dentin (9). Interestingly, usage of “all-in-one” adhesive system has also shown to halve the total treatment duration (10,11). The adhesion is less affected following salivary contamination while using self-etching primers than the conventional phosphoric acid etchants, because of the acidic nature of the former (12-14).

Prevent Seal (Itena) is one of the latest self-etching sealants that has been introduced in the dental market. The one step application and fluoride releasing properties of this sealant as claimed by the manufacturers can be added advantages of prevent seal (https://www.itena-clinical.com/en/care-prevention/49-preventseal.html). However it’s important to study the physical properties of prevent seal, as they may differ from that of the conventional resin sealants (1). Thus, the present study was conducted to comparatively evaluate sealing ability, penetration and adaptation of a self-etching pit and fissure sealant and a conventional resin sealant. The null hypothesis was set as there will not be any difference in the sealing ability, penetration and adaptation of a self-etching pit and fissure sealant, when compared to that of the conventional sealant.

## Material and Methods

This *In vitro* Experimental Intergroup comparative study was initiated after approval from Institutional Ethics Committee.

Study setting and population: The study was conducted on non-carious erupted, young, immature permanent teeth (molars and premolars) extracted for orthodontic and therapeutic purposes.

Sample size.

**Figure 1:**
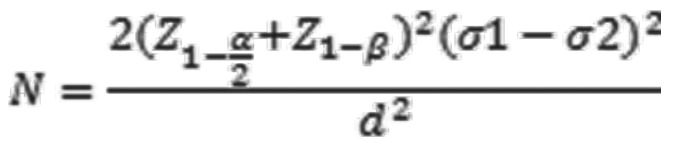
Formula.

Based on the study report by Savi E *et al.* (https://dvd-dental.com/media/attachments/FT-PreventSeal-ENG.pdf) from Marseilles university, the Clinpro and Prevent Seal groups had a standard deviation of 15 and 23 respectively in relation to the % of sealing capacity. With 5% alpha error (having Z alpha of 1.96), 80% power (Z beta of 0.84) for the study and a clinically significant difference of 28 units, the required sample in each group was derived to be 8 in each group. The formula used for sample size calculation was (Fig. 1):

Procedure: The crown portions of the selected teeth were cleaned using a water slurry of fine pumice and a slowly rotating rubber cup. After thorough rinsing and air-drying, a total of 32 teeth were then randomly and equally divided into two groups of 16 teeth each-

Group I (Conventional acid etch fissure sealant): The entire fissure region was first etched with 37% phosphoric acid gel (Scotchbond Etchant, 3M ESPE) for 30 seconds. This was followed by a water rinse for 20 sec and drying with oil free compressed air. A conventional fissure sealant (Clinpro sealant, 3M ESPE), was then applied according to manufacturer’s instructions. Group II (Self-etching sealant): Self etching sealant Prevent Seal (Itena, North America) was directly applied on cleaned pits and fissures without prior etching according to manufacturer’s /instructions.

The sealant material applied on teeth belonging to both the groups were allowed to penetrate into the fissure for 20 sec and polymerized using a visible light curing unit (3M, United States) for 20 sec.

Phase 1: Evaluation of sealing ability-

Following sealant application, 16 teeth (8 teeth per group) were subjected to thermal cycling for 1,500 cycles alternating between 5°C and 55°C. The apex of each tooth was sealed using sticky wax following which 2 layers of nail polish was applied all over except the sealant and 1 mm of its margin. The teeth were then immersed in 1% methylene-blue solution, buffered at pH 7 for 24 hours, following which they were rinsed and cleaned under running water for 10 minutes.

**Figure 2:**
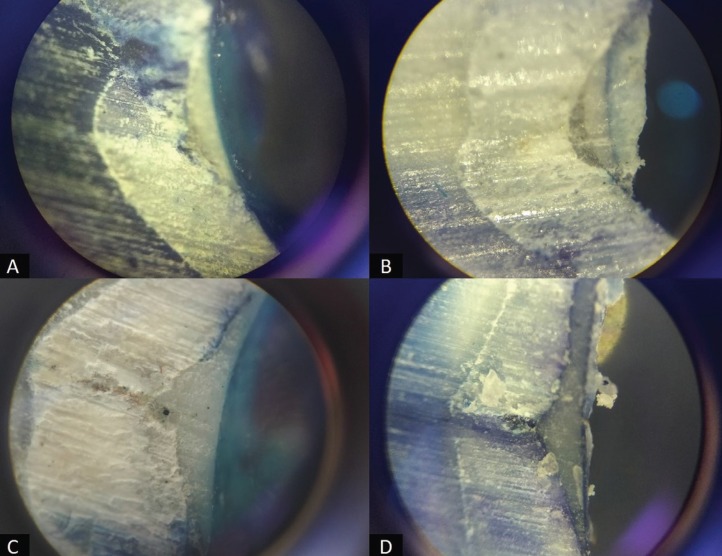
Different microleakage scores as seen by stereomicroscopic examination: A) Score 0, B) Score 1, C) Score 2, D) Score 3.

The teeth were later ground against a lathe to obtain mesio-distal half of the tooth. Dye penetration of obtained tooth section was examined under CH20 Olympus microscope with a NA at x40 magnification. Each section was photographed and then evaluated using the criteria given by Övrebö and Raadal (15) (Fig. 2A-D)

Score 0: No penetration of the dye seen in the section

Score 1: Penetration into the part around the sealant

Score 2: Penetration into the part below the sealant

Score 3: Penetration at the base of the fissure

Phase 2: Evaluation of sealant penetration and adaptation-

**Figure 3:**
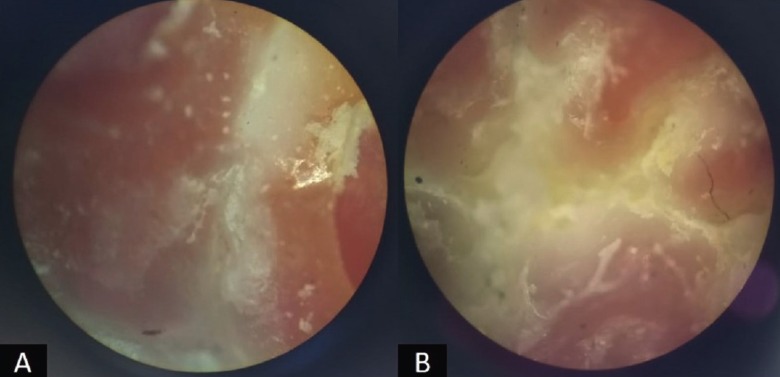
A) Bubbles and debris visible on examining with stereomicroscope and B) Tags present on the bottom and slopes of the fissure.

Remaining 16 sealed teeth (8 samples in each group) were immersed in 30% nitric acid solution for 6 hours to get dissolved and to obtain only the sealants. The base of thus obtained sealant served as the replica of the fissure. The replicas were rinsed with deionized water and mounted on a slide. Stereomicroscopic analysis was done to check sealant penetration and adaptation properties (Fig. 3A-B) viz:

a) Bubbles in the bottom of fissure

b) Debris in the fissure 

c) Tags in the bottom of the fissure

d) Tags at cuspal slopes and fissure entrance

Out of the sixteen samples used for stereomicroscopic analysis of sealant penetration and adaptation, 8 samples (4 in each group) were randomly selected. The etching pattern of the selected samples was analyzed using Scanning Electron Microscope (SEM) (Carl Zeiss, Germany). The etching pattern were categorized according to Silverstone *et al.* (16) as.

Type 1: Enamel prism cores preferentially removed.

Type 2: Reverse pattern where the peripheral regions of the prisms were removed leaving relatively unaffected prism cores.

Type 3: Areas corresponding to both Types 1 and 2 present.

Two trained observers independently did the microscopic examination and scoring. It was decided to take third observer’s opinion, in the presence of inter-observer disagreement. However, no inter-observer differences were noted during scoring procedure.

Statistical analysis:

The presence or absence of bubbles, debris, tags as well as scores of microleakage and pattern of etching were compared between the two groups using Fishers Exact modification of Chi Square test.

## Results

**Figure 4:**
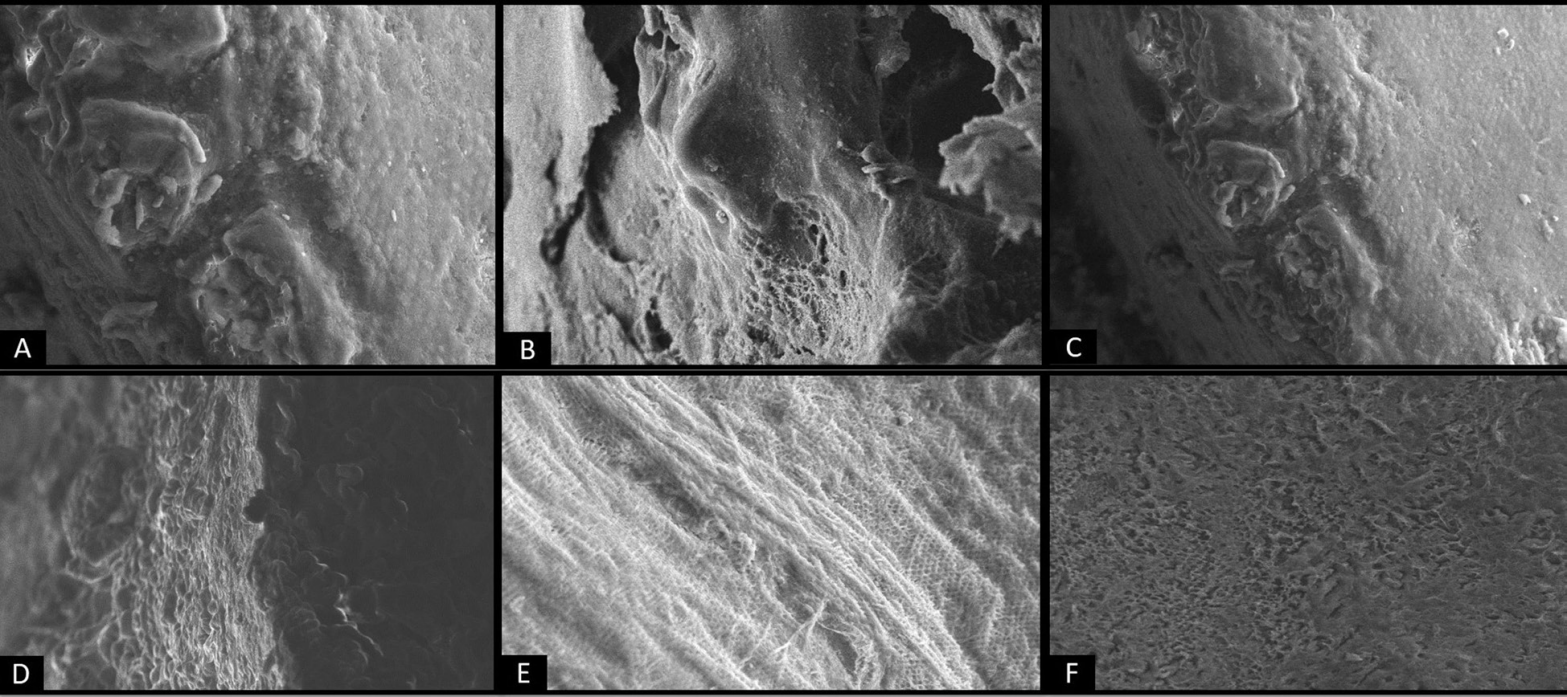
A-C) Etching patterns seen in Group I under Scanning Electron Microscope- A) Type I (magnification X 1.00K), B) Type II (magnification X 1.00K), C) Type III (magnification X 600K), D-F) Etching patterns seen in Group II under Scanning Electron Microscope- D) Type I (magnification X 1.00K), E) Type II (magnification X 600K), F) Type III (magnification X 600K).

Microleakage while using conventional acid etch sealant vs self-etching sealant was not statistically significant ( Table 1). All the scores (score 0, 1, 2, 3) were diversely distributed among the two groups. When group I and Group II were compared in relation to different aspects of sealant penetration and adaptation, there were no statistically significant differences observed viz Bubbles in the bottom of the fissure (*p*=0.131), Debris in the fissure (*p*=0.131), Tags in the bottom of the fissure (*p*=0.302) and Tags at cuspal slopes and fissure entrance (*p*=0.106). However, when the presence of bubbles in the bottom of the fissure was compared between two groups, 75% of Group I samples had porosity as against only 37.5% of Group II. Similarly, 62.5% of group I samples contained debris in the fissure against 25% of group II samples. 75% and 50% of Group II samples showed the presence of tags at the bottom of the fissure and cuspal slopes/ fissure entrance respectively, while the same was seen only in 50% and 12.5% of group I samples. On comparing the etching pattern as seen under SEM (Fig. 2), no specific pattern was seen in any of the two groups, but a diversity of distribution was seen (Fig. 4A-F) (Chi square value = 0.667 and p value= 0.717).

**Table 1:**
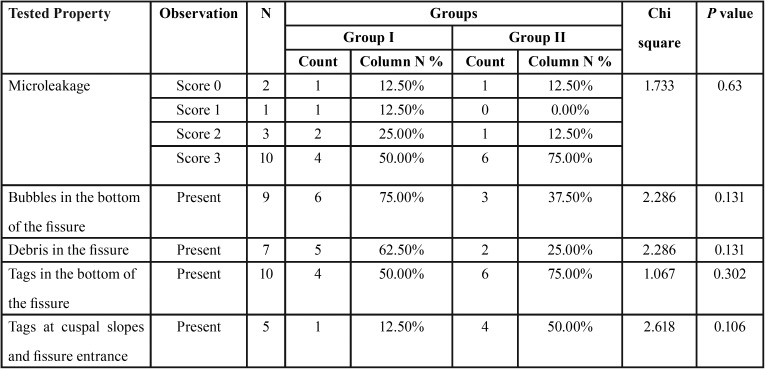
Comparison of tested properties between two groups as done using Chi square test.

## Discussion

Perfect isolation and precise technique are critical factors responsible for sealant retention (17). Thus, considering the patient behavior and compliance in pediatric dentistry, short sealant placement procedure with minimal steps are more desirable (6). Aiming at this need, the latest development in pit and fissure sealant category, is the “self-etching sealant” of which Prevent seal is the one, which negotiates etching. Sealant placement is thus a simplified one step procedure, where in the clinician directly places the sealant on cleaned pits and fissures and cures it. However, it can be a boon to preventive dentistry only if it provides good seal and retention. Microleakage at the sealant margins, may predispose the tooth to dental caries (6). Thus, in the present study we comparatively evaluated the sealing ability, penetration and adaptation of a self-etching pit and fissure sealant- Prevent Seal with that of a most commonly used conventional resin sealant- Clinpro.

In the present study, we followed the conventional technique of sealant placement wherein tooth was cleaned, etched and sealed. The application of bonding agent after etching is still a controversy. The critics of the same put forth this as an time consuming additional step, which also increases the treatment cost (18). Long term follow up clinical studies by Mascarenhas *et al.* (19) and Boksman *et al.* (20), concluded that use of bonding agent before sealing does not improve the sealant retention in the long run. Though, few other studies (21-24) reported significantly less microleakage scores when bonding agent was used before sealing, considering the added treatment time and possible isolation breach in young-uncooperative children, this technique is more feasible and acceptable in older patients (18).

Results of present study showed no statistically significant difference in the microleakage scores between two groups. This is in well agreement with the findings of a study by Nahvi A *et al.* (18), who compared self-etching sealant (Prevent seal) with conventional acid etch sealant with / without application of bonding agent. Findings of the later study showed prevent seal had comparable microleakage scores to conventional acid etch sealant (Clinpro) when applied without bonding agent. A study by Jabbarifar SE *et al.* (25), also showed no statistically significant difference in the microleakage between Prevent Seal self-etching fissure sealant, Clinpro conventional fissure sealant and Filtek Flow flowable composite resin. On the other hand, Parco TM *et al.* (6) reported significantly higher microleakage scores for self-etching sealant (Enamel Loc) than that of conventional acid etch sealant (UltraSeal XT Plus), irrespective of the contamination conditions. This contrast finding can be attributed difference in the types of included teeth, types of sealants used and study methodology.

In the present study, when sealant penetration and adaptation properties were evaluated, greater percentage of group I samples had bubbles in the bottom of fissure and debris in the fissure. When tags in the bottom of the fissure and at cuspal slopes/ fissure entrance were compared greater percentage was noted in group II. However, these differences were not statistically significant. The observed differences could be attributed to low viscosity of prevent seal which enables deeper fissure penetration (18). To best of our literature search, this study is one of its kind, which evaluated the sealant penetration and adaptation properties of a self-etching sealant. Existing studies on self-etching sealants have evaluated mostly microleakage (6,18,25). When etching pattern was evaluated, a diverse pattern was observed among the samples of both the groups. This observation was inconclusive owing to small number of samples subjected to SEM.

Results of our study were promising to make use of self-etching sealant prevent seal, especially considering the compliance and limited treatment time available for a pediatric patient. Also, novel features like 21-MPa retention with the enamel, release of fluoride, greater flow and simplified application have the potential to make any clinician choose a self-etching sealant prevent seal over the conventional acid etch sealant (18). However, future studies are recommended evaluating the retention and efficiency of prevent seal in preventing caries under in vivo conditions. Also, SEM studies evaluating the etching pattern are also recommended on a larger sample size.

## Conclusions

Within the limitations of the present study, the self-etching sealant Prevent seal was found to have similar microleakage, sealant penetration and adaptation properties as conventional acid etch sealant.
